# Treating Sexual Orientation Obsessive-Compulsive Disorder with Cognitive Analytic Therapy: Case Report and Quasi-Experimental Outcome Evaluation

**DOI:** 10.3390/reports8020051

**Published:** 2025-04-17

**Authors:** Ese-Oghene Osivwemu, Melanie Simmonds-Buckley, Chris Gaskell, Stephen Kellett

**Affiliations:** 1 Department of Clinical and Applied Psychology, University of Sheffield, Sheffield S1 2LT, UK; 2 Inpatient Wards, Rotherham Doncaster and South Humber NHS Foundation Trust, South Yorkshire S26 4TH, UK; 3 Neuropsychology Department, North Staffordshire Combined Healthcare NHS Trust, Stoke-on-Trent ST4 8HH, UK

**Keywords:** sexual orientation obsessive–compulsive disorder, cognitive analytic therapy, case report, single-case experimental design

## Abstract

**Background and Clinical Significance**: Evaluations of interventions for sexual orientation obsessive–compulsive disorder (SO-OCD) are rare. This study therefore evaluated the effectiveness of cognitive analytic therapy (CAT). **Case Presentation**: A 28-year-old heterosexual male presented with SO-OCD in the form of obsessions concerning his sexual identity and associated avoidance and reassurance-seeking compulsions. The evaluation was a quasi-experiential A/B single-case experimental design (SCED) with follow-up of the eight-session CAT intervention. The SCED had three phases: baseline ‘A’ (two sessions; 21 days), active treatment ‘B’ (six sessions; 56 days) and follow-up (one session; 44 days). There were seven daily rated idiographic outcome measures (intrusion count as the control, a compulsion count and then intensity measures of checking, worrying, generating evidence, shame and anxiety). Four nomothetic outcome measures (including a primary outcome measure of SO-OCD) were collected at assessment, end of treatment and follow-up. Generalised logistical models were fitted to the idiographic outcomes. Six of the seven idiographic measures responded to treatment, indicating an effective intervention. Idiographic change was non-linear and synchronised with specific psychotherapeutic actions and there was no evidence of relapse. There was a clinically significant and reliable pre–post reduction in SO-OCD with progress sustained over the follow-up period. **Conclusions**: Overall, the study indicates that CAT was an effective intervention for the SO-OCD. The study methodology is critiqued and guidance on SO-OCD treatment is provided.

## 1. Introduction and Clinical Significance

The four main symptom dimensions [1] of obsessive–compulsive disorder (OCD) are contamination, doubt/harm, perfectionism (symmetry/ordering/exactness) and forbidden/taboo thoughts. When the focus of the sexually taboo thoughts concerns sexual orientation, the diagnosis is one of sexual orientation obsessive–compulsive disorder (SO-OCD; [2,3,4,5]). Lifetime prevalence of SO-OCD has been estimated to vary between 9.9% [6] and 11.9% [7], with SO-OCD affecting more males than females [7]. SO-OCD is understudied and infrequently presents in services, and when it does, it is then commonly misdiagnosed as some form of sexual identity crisis [7].

The evidence base for both the assessment and treatment of SO-OCD is therefore piecemeal and there is a lack of any clinical trial evidence. Assessment has benefitted from development of the valid and reliable self-report 14-item *Sexual Orientation Obsessive–Compulsive Scale* (SO-OCS; [8]). The evidence base for intervention consists of just three studies, and these studies are methodologically weak due to either being largely qualitative case reports or the study being conducted in a student sample. Fluoxetine and behaviour therapy (specifically, exposure and response prevention; ERP) were combined to treat a 20-year-old male heterosexual SO-OCD case. The Yale Brown Obsessive Compulsive Scale (Y-BOCS) score fell from 28 pre (severe) to 8 post (mild) and the case was symptom-free at 20-week follow-up [9]. When SSRIs were used a treat a heterosexual 51-year-old male SO-OCD patient, there was little effect [4], but the introduction of ERP enabled change; the Y-BOCS fell from 24 (severe) to 3 (post) and 4 (follow-up). When self-help association splitting techniques were compared to a waitlist control condition in N = 120 heterosexual participants (in a non-clinical and student sample), both groups had reductions in semantic associations for the sexual obsessions, thought suppression and SO-OCD symptoms [2]. There is therefore a gap in the evidence base in terms of well-controlled evaluations of interventions in clinical populations. 

The evaluation methodology of scientific choice when a treatment evidence base is thin (i.e., as is the case with SO-OCD) is first to conduct a single-case experimental design (SCED; [10]). SCED relies on intensive idiographic and nomothetic measurement and associated analysis of change across and between prespecified study phases. Any change in idiographic outcomes is more convincing when it is plotted against a control idiographic variable that is not the target of the intervention and so therefore not predicted to change. Previous SCED research has also assumed linear effects and/or instant effects of treatment in idiographic measures and this unfortunately creates the risk of statistical assumptions being violated [11]. There is a large body of evidence demonstrating that change during psychotherapy is more curvilinear [12]. Thus, contemporary SCED practice suggests adopting more advanced statistical methods, such as generalised logistical models (GLMs), to plot non-linear, non-instantaneous therapeutic change over the phases of a study [11]. The study hypotheses were as follows: (a) change would be gradual and non-linear during the intervention compared to baseline on a range of idiographic SO-OCD measures, (b) reductions in the compulsion count idiographic measure over treatment time would be found in relation to stability in the intrusion count (i.e., on the control idiographic measure), (c) there would be no evidence of relapse in the idiographic measures and (d) there would be clinical and reliable reduction in the SO-OCS nomothetic primary outcome measure that was maintained at follow-up.

## 2. Case Presentation

The patient was a 28-year-old cisgender single male who lived in shared accommodation and was employed in an administrative role. The patient had previously been assessed by his General Practitioner and given the diagnoses of comorbid OCD and depression. It was considered that the comorbid presenting problem was moderately severe. The patient had never been seen by a psychiatrist. Throughout the present study, the patient was prescribed and had been taking Sertraline medication for 4 years previously (50 mg daily). The patient considered the Sertraline to be helpful, but that the medication had never enabled recovery from OCD and the symptoms had remained stable but worsened at times of heightened stress. He had been referred to an Improving Access to Psychological Therapies (IAPT) service aged 20 and received an ineffective low-intensity worry management guided self-help intervention. The patient also had sought private cognitive behaviour therapy (CBT) aged 24 and he described this as ineffective. There was no information available on the competency of these previous psychological interventions. Age of onset of the SO-OCD was at 18 years and overlapped with the start of university (humanities degree) and the potential development of close male friendships. Cognitive analytic therapy (CAT) was offered because of the previous non-response to low- and high-intensity CBT, the relational nature of SO-OCD and CAT being a relational psychotherapy.

Throughout his adult life, the patient complained of distressing and high-frequency intrusions concerning doubts regarding his heterosexuality that created intense feelings of shame, anxiety and guilt. The intrusions would typically consist of doubt-type thoughts (e.g., ‘what if am gay?’) but would also occasionally be images (e.g., an image of himself engaging in gay sex). He denied any urge-type impulsions (e.g., any persistent and strong impulses to have gay sex). He held a belief and an associated fear that he would wake up one day and suddenly find out that he was gay, and he also would worry about what other people would make of this apparent sudden shift. To reduce the shame, anxiety and guilt, he had become dependent on a range of overt and covert compulsions and had a raft of avoidant behaviours. In terms of covert compulsions, the patient would interoceptively check his sexual physiological response when in the company of men, analyse and check for feeling romantically attached to any males, analyse/check/amend his behaviour for any interpersonal signs of being ‘camp’, and constantly reassure himself that he was heterosexual. He would worry about whether he had looked at another man for too long (e.g., attractive male models in advertisements). In terms of overt compulsions, the patient would try to always act in a ‘macho’ manner and use heterosexual pornography to test his physiological response in the effort to prove to himself that he was heterosexual. The patient would panic watching heterosexual pornography if he lost his erection, or if he felt he was looking too much at the male actors. In terms of avoidant behavioural patterns, the patient reported not allowing close relationships with male friends/colleagues to develop, avoiding sexual relationships with women for fear of sexual failure and avoiding visual contact with physically attractive male figures in films, television and print media.

The patient was raised in a nuclear family with one male sibling. The relationship with the father was described as difficult as a child, due to the father being a hyper-masculine and very critical authority figure. The patient stated that he always felt in his father’s shadow and was a disappointment to him and was often belittled for his intellectual and physical lack of prowess. He reported that any achievement would be undermined. He described an emotionally enmeshed relationship with his mother, as he sought emotional closeness. The patient was bullied at primary school due to being quite feminine and took the decision on the transition to secondary school to change his interpersonal style. This involved pretending to be more interested in sports, only having male friends and behaving in a more macho manner. He described a lifelong schism between his behavioural public self (i.e., macho, hard and masculine) and felt private self (i.e., insecure, soft and feminine). When the patient attended university, he struggled with loneliness due to distancing himself from male friendships. The patient stated that he was depressed throughout university and had struggled with depression periodically since. He had a few short-lived heterosexual relationships. The patient was not in a relationship during the study. The diagnosis of SO-OCD was confirmed by the SO-OCD score (see measures section).

### 2.1. Evaluation Design

The study follows the single-case reporting guideline (SCRIBE) statement to ensure accurate reporting [13]. The quasi-experiential A/B design had an additional follow-up (FU) phase and so the study design was therefore A/B-FU. The first phase (baseline: A) lasted for 21 days and encompassed 2 assessment sessions. The second phase (B) lasted for 56 days and contained 6 treatment sessions. The follow-up phase lasted for 44 days and had one session at the culmination of that phase. The study therefore had a 121-day timeline.

### 2.2. Treatment

The intervention was 8 sessions of cognitive analytic therapy (CAT; [14]). CAT is a time-limited, integrative, relational and transdiagnostic therapy delivered in 8-, 16- or 24-session formats that is now practiced internationally [14]. CAT is based on a theoretical foundation that mental representations of self and others during adult life have been developmentally shaped by early experiences and interactions during childhood and adolescence, and these will be evident (and need to be managed) in the therapeutic relationship. In CAT, presenting problems or diagnoses are redefined relationally during narrative and diagrammatic reformulation as ‘target problems’ (TPs) and associated ‘target problem procedures’ (TPPs). TPPs are labelled as either traps (vicious circles), snags (self-sabotage) or dilemmas (false negative options). Reciprocal roles summarise how adverse childhood experiences are internalised and then influence here-and-now self-to-self, self-to-other and other-to-self interactions. The cognitive component of CAT are the relational patterns and procedures, and the analytic aspect of CAT are represented by reciprocal roles. CAT involves three interrelated stages of reformulation, recognition and revision [14,15]. The first phase (reformulation) involved reformulating the presenting problem (e.g., via narrative and sequential diagrammatic reformulations; SDR). The second (recognition) phase involved the scaffolding of enhanced recognition of reciprocal role activation and associated patterns and procedures (e.g., via ‘homework’ self-monitoring and in-session analysis). The third and final (revision) stage involves use of active change methods called ‘exits’. The clinical competencies during each of the three phases have been summarised in the CAT competency model [15]. The therapist was a male cisgender (heterosexual) Consultant Clinical Psychologist and CAT psychotherapist under monthly clinical supervision. The therapist therefore had completed 4 years of post-doctoral training in CAT and was also a CAT trainer and supervisor. At the time of completing the research, the therapist had been qualified as a CAT practitioner for over 20 years. All weekly sessions were attended and lasted for 50 min.

The first two sessions were focal to assessment tasks, did not contain any treatment elements and culminated in a narrative reformulation (i.e., read at the start of session three). The patient was asked to also write a reformulation on himself for session 3 (using written guidance) and common elements were integrated into a final and collaboratively agreed version. A common element across the written work was the role of the father in undermining the patient and inducing anxiety—and in the therapist’s reformulation, this undermining was linked to the constant doubting of sexuality. The narrative reformulation from the therapist therefore made links between the past and present, named potential enactments in the therapeutic relationship (e.g., the therapist getting drawn into being over-caring, the patient being undermining of the help offered by the therapist and the patient feeling undermined rather than appropriately challenged; see below for the reciprocal role summary) and stated the TPs and TPPs. The narrative formulation therefore contained the following TP and TPPs consisting of two traps (TP1 and TP2) and one dilemma (TP3). TP1: Relationships with women. TPP1: I feel insecure in my sexuality, and so I avoid allowing relationships with women to develop and deepen for fear of being undermined and humiliated, and so I continue to feel insecure in my sexuality. TP2: Fear of being gay. TPP2: Always feeling unsure of myself, I have latched onto a fear that I might be gay. I am plagued by thoughts telling me that I am gay or doubting that I am not gay. This makes me set tests for myself to pass to try to prove to myself that I am heterosexual. I pass the test, but then another thought happens and this creates more uncertainty. TP3: Either/or sexuality. TPP3: I am either completely heterosexual or I am completely gay. The delivery of the narrative reformulation was considered the end of the baseline and the start of active treatment, as is consistent with previous CAT SCED research [16]. The SDR was initially sketched during session 3 and was fully completed at session 4. The SDR contained two reciprocal roles, over-caring to dependent and undermining to unsure, with the TPPs from the narrative reformulation presented in visual form and used them to connect the reciprocal roles. The recognition work consisted of the patient keeping a narrative diary of when TPPs were activated and a tally chart of when he had been in the top or bottom of a reciprocal role. TPP recognition sheets were used after session 3 to review progress in the development of awareness.

The ‘exits’ added to the SDR to support change during the revision phase were as follows: (a) exposure to intrusions and response prevention to compulsions, (b) developing closer relationships with male friends, (c) starting to exercise, (d) investing in rather than undermining life goals and projects, and (e) starting to place trust in oneself. The patient stated that because the rationale of ERP had been explained more clearly than during CBT and that because he now understood the intrusions as being based in the relationship with his father, he could now successfully allow exposure to the intrusions to occur. ERP was therefore completed in sessions 5, 6 and 7 and also was a homework task to ensure repetition. CAT is also a therapy that actively works with enactments in the therapeutic relationship [14]. The most common enactments were analysis of when the patient felt undermined by the therapist (and vice versa), when reassurance-seeking occurred and when the sessions felt like ‘perfect care’ was occurring. In terms of treatment fidelity, the hallmark components of CAT are a narrative reformulation, an SDR and goodbye letters exchanged at the termination of therapy by patient and therapist [15]. In the current case, all these distinctive features of the CAT model were present.

### 2.3. Idiographic Measures; Description and Analysis

Seven idiographic measures (2 frequency and 5 intensity measures) were co-created in the first assessment session. The first measure was a daily intrusion frequency count, and the second measure was a daily count of compulsions. The intrusion count was designed as a control idiographic measure [10]. This measure was not expected to change due to the intervention, and so the relative change in other measures could be evaluated against anticipated stasis in the control measure. The third, fourth and fifth measures were drawn from the Sexual Orientation Obsessive–Compulsive Scale [8], adapted to fit daily measurement. The third measure was “I have been checking myself physically and mentally today to ensure that I am not sexually attracted to men”, the fourth measure was “I have been worrying today that I am gay and in denial”, and the fifth measure was “I have been generating evidence to prove I am a heterosexual man today”. The sixth measure was a measure of shame (i.e., “Today, I felt ashamed”) and the seventh a measure of anxiety (i.e., “Today, I felt anxious”). Measures 3–7 were all rated on a Likert scale rated from 0 (not at all) to 9 (all the time). As these daily measures were idiographic, there is no psychometric reliability and validity information as this type of measure does not lend itself to formal psychometric evaluation. As the measures were co-designed, they had high face validity for the patient.

Kendall’s Tau (τ), a non-parametric correlation, tested for the presence and strength of within-phase trends during the baseline phase. Three nonoverlap statistics were calculated to supplement visual analysis of idiographic time series graphs and compared the degree of differences in paired phases (A vs. B and A vs. FU). Percentage exceeding the median (PEM; [17]) established the proportion of data points above the median of the baseline in subsequent phases and was interpreted as follows: a PEM score above 0.9 suggested a highly effective intervention, between 0.7 and 0.9 suggested a moderately effective intervention and <0.7 suggested an ineffective treatment [18]. Nonoverlap of all pairs (NAP; [19]) established the percent of all pairwise comparisons where the intervention (B) and follow-up (FU) phase data points were greater than baseline (A) data points, with exact pairs gaining a weight of ½. A PEM score less then 0.65 would indicate a weak treatment effect, a PEM score in the range of 0.66–0.92 would indicate a medium treatment effect, and a PEM score greater than 0.93 would indicate a strong treatment effect [19]. Percentage of all nonoverlapping data (PAND) calculated the proportion of observations remaining after removing the minimum number of observations required to remove all overlapping data in the compared phases [20]. A PAND score of >0.90 indicates a very effective treatment, between 0.70 and 0.89 indicates moderate effectiveness, 0.50–0.69 indicates a questionably effective treatment, and <0.50 indicates that treatment is ineffective [21].

Generalised logistical models (GLMs) using an optimisation function were fitted to the A vs. B phase comparisons to allow non-linear changes to be modelled, and thus to identify where and when change started and finished [11]. The generalised logistic function estimates the curvilinear effect in model parameters and so presents the time during treatment at which the rate of change is at its largest (i.e., where the curve is at the midpoint); this is known as the *inflection point*. The growth rate specifies how steep the curve is (i.e., how fast change is occurring). This is calculated as the average rate of change on the idiographic measure between each phase. The growth rate is estimated between −2 and +2, with higher values indicating faster rates of change. Further metrics such as R^2^ and ES_r_ (the proportion of scale improvement considering floor and ceiling effects: [22]) were computed to allow contextualisation with the common effect size for Cohen’s D (ES_c_; [11]). GLMs were visually plotted to illustrate the rate and non-linear shape of change and the inflection points.

### 2.4. Nomothetic Measures: Description and Analysis

Four nomothetic self-report measures were taken at session 1 and session 8 and at follow-up. Nomothetic outcomes were analysed using the reliable change index [RCI; [23]] and where clinical norms were available, clinical change rates were calculated. RCI assesses whether the degree of change on a measure is beyond random measurement error or chance, and an RCI value ≥ 1.96 indicates significant and reliable change [23]. Nomothetic measures were also assessed for clinical change (i.e., when scores shifted from a clinical to a non-clinical population). There are four categories of patient outcomes using reliable and clinical change criteria when the intake score score meets caseness: recovered, improved, unchanged and deteriorated [23].

*The Sexual Orientation Obsessive–Compulsive Scale* [*SOO*-*CS*: [8]] is a 14-item questionnaire that assesses sexual orientation obsessions and compulsions. Items are rated on a 5-point Likert scale from 0, ‘not at all’, to 4, ‘extremely’, with a total score ranging from 0 to 84. The clinical cut-off is a score of 18+. The SO-OCS has very good internal consistency (Cronbach’s alpha = 0.90) and excellent sensitivity (98.1%) and specificity (99.7%). The SO-OCS was the primary nomothetic outcome measure for this study.

*The Patient Health Questionnaire-9* (*PHQ-9*; [24]) comprises 9 items that assess the corresponding criteria of symptoms for major depressive disorder (MDD) according to the DSM-IV [25]. The PHQ-9 uses a 4-point Likert scale, with total scores ranging from 0 to 27. Scores are categorised as no depression (<4), mild (5–9), moderate (10–14), moderately severe (15–19) or severe (20–27). The clinical cut-off is ≥10. The PHQ has excellent reliability (Cronbach’s alpha = 0.86–0.89) and good sensitivity (80%) and specificity (88%).

*The Quality-of-Life Enjoyment and Satisfaction Questionnaire—Short Form* (*Q-LES-Q-SF*; [26]) is a 16-item tool that assesses enjoyment and satisfaction in everyday life. Responses are graded on a 5-point Likert scale from 0 to 5 and total scores range between 0 and 70. The greater the total scores, the higher the quality of life, enjoyment and satisfaction. The Q-LES-Q-SF has an excellent internal consistency (Cronbach’s alpha = 0.90), 100% specificity and good sensitivity (80%). The measure was selected to assess the generalisability of the CAT intervention.

*The Obsessive-Compulsive Inventory* (*OCI*; [27]) is a 42-item self-report measure of common obsessions and compulsions. Scores are rated on a 5-point Likert scale (0–4) for occurrence of distress on each item, with a score range of 0–168. The higher the score, the more severe the OCD. The OCI has good internal consistency (Cronbach’s alpha = 0.92 and 0.93) and adequate test–retest reliability.

### 2.5. Statistical Software

Statistical analyses and visualisations were conducted using R (R Core Team, 2020, v 4.0.2). Nonoverlap tests were calculated using ‘SingleCaseES’ (version 0.7.3.9999) [28] while regression analyses and plots were developed using ‘userfriendlyscience’ [29].

### 2.6. Results

**Figure 1:**
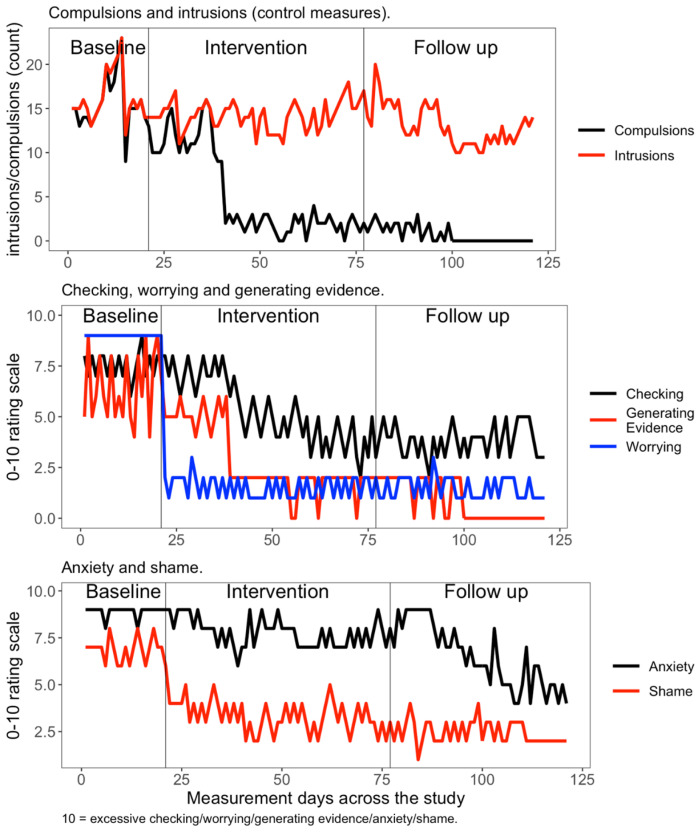
Time series plots for idiographic outcomes per phase of study (A/B-FU).

Results are divided into four sections to address the study hypotheses: idiographic time series plots, idiographic nonoverlap analysis, idiographic GLM analysis (i.e., A/B comparison only) and nomothetic outcomes. Time series plots for each idiographic measure for each phase of the study are provided in Figure 1. Kendall’s Tau (τ) demonstrated that there was no significant baseline trend for intrusions (τ = 0.06, *p* = 0.74), checking (τ = 0.06, *p* = 0.79), worrying (τ = 0.00, *p* = 1), generating evidence (τ = 0.11, *p* = 0.53), feeling ashamed (τ = −0.04, *p* = 0.86) or feeling anxious (τ = 0.04, *p* = 0.9). There was a significant baseline trend (i.e., indicating weak improvement prior to treatment starting) on the compulsion count (τ = 0.12, *p* = 0.49).

The control idiographic measure of intrusion frequency remained stable across all phases of the study. The narrative reformulation was associated with immediate reductions in worrying and feeling ashamed, and these gains were then maintained over treatment and then follow-up. The reduction in compulsions, the intensity of the checking and the generating evidence idiographic measures all corresponded to the introduction of ERP. Trend lines (not reported in the graphs) showed continued reductions over follow-up time for each idiographic measure, except for checking and worrying, which both plateaued. The anxiety idiographic outcome measure changed the most during the follow-up phase.

Table 1 presents the grand phase means (SDs) for the idiographic measures. This demonstrates reductions during treatment compared to baseline and that gains were maintained over follow-up. TAU, PEM, NAP and PAND results are presented in Table 2. Interpretation of the PEM and NAP results for the comparison of all phases suggests CAT was a highly effective intervention across all measures (i.e., bar the intrusion count control measure). PAND analysis showed treatment (compared to baseline) as moderately effective for reduced checking, generating evidence and anxiety. PAND baseline to follow-up analysis suggested CAT was highly effective for all measures except for intrusions and anxiety, which were moderately effective.

**Figure 2:**
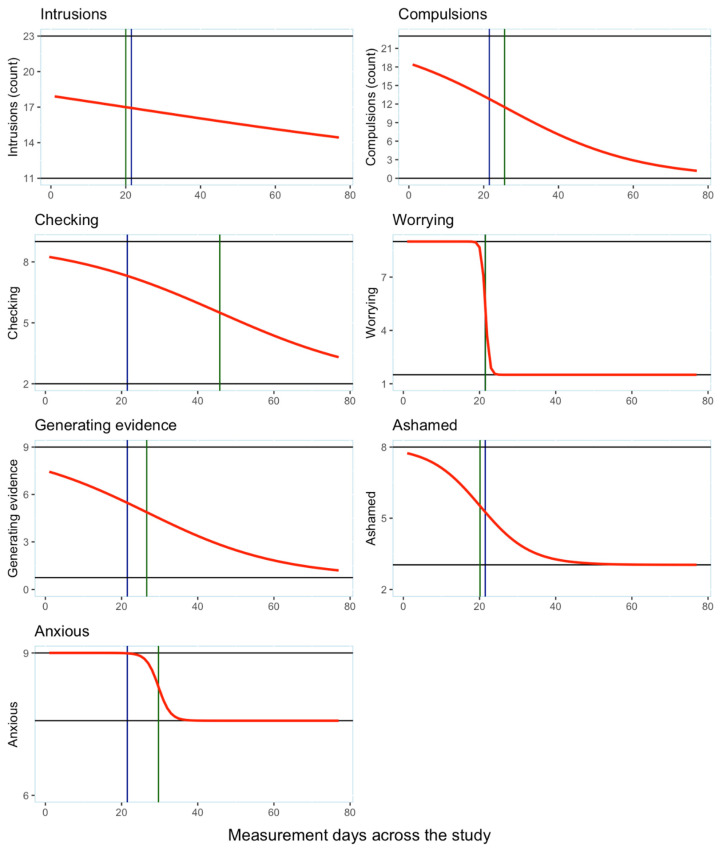
GLM analysis of change in idiographic outcomes. Vertical blue line: the start of treatment; vertical green line: the inflection point (IP): horizontal lines: the floor and ceiling values.

In the GLM figures (see Figure 2), the vertical blue line indicates the start of treatment, the vertical green line indicates the inflection point (IP) and the horizontal lines represent the floor and ceiling values. These indicate that the decrease in the intrusion count and shame occurred slightly before the introduction of the treatment phase. The inflection point for reduced worrying coincided with the narrative reformulation, slightly following narrative reformulation for compulsions, generating evidence and anxiety and nearly midway through treatment for the checking measure. Examination of growth rates (see Table 3) shows the quickest improvement was for reduced worrying (i.e., indexed by an almost vertical line), followed by reduced anxiety and shame. The compulsion count, intensity of checking and intensity of generating evidence showed more gradual improvements over the course of treatment.

The GLM did not summarise the data well for the intrusion count measure as reflected by the negative R^2^ value (see Table 4). Similarly, the anxious measure also had poor fit. However, the model appeared to be a better fit for the compulsion count, checking, generating evidence and shame measures. The worrying measure appeared to fit the model best due to the curvilinear shape in the graph as reflected in the reported R^2^ value. Examination of the effect sizes (i.e., the ESc and ESr values in Table 4) shows that treatment had the least effect on anxiety and the greatest effects on compulsions, checking, worrying, generating evidence and shame. The greatest intervention effect was indicated to be on the intrusion count measure (shown by the ESc effect size) and this was in contrast to the flat graphical representation of the control measure data.

The nomothetic outcomes are presented in Table 5. In terms of severity at assessment, the PHQ-9, OCI and SO-OCS were all in the clinical range. There was a reliable and clinically significant reduction in pre–post SO-OCD scores, with progress maintained at follow-up. This would constitute SO-OCD recovery. Moderate depression was apparent at assessment and this changed to mild depression at end of treatment and follow-up. This represents a pre–post reliable and clinically significant reduction in depression. Scores on the general OCD measure (OCI) were just above the cut-off for a clinical case; no reliable change occurred in the OCI measure, but the termination score was in the community range. There was a reliable pre–post increase in Q-LES-Q-SF life enjoyment and satisfaction (i.e., the nomothetic measure of generalisation), with minimal evidence of any relapse occurring. No adverse effects occurred.

## 3. Discussion

This study aimed to empirically and thoroughly evaluate outcomes in a rare clinical case of SO-OCD treated with CAT. The A/B-FU design evaluated effectiveness in a case which had previously been unresponsive to CBT. There has been limited evaluation of the effectiveness of CAT for OCD previously. A single previous pilot clinical trial (N = 20) showed that CAT was differentially effective when compared to waitlist control [31]. The current study suggests that when idiographic and nomothetic outcomes were considered in tandem, the presenting problem of SO-OCD was effectively treated, and that the positive change was maintained over follow-up time. All sessions were attended, suggesting an acceptable therapeutic approach. There was a reliable and clinically significant reduction in SO-OCD and depression on the nomothetic outcome measures, and the patient’s quality of life (Q-LES-Q-SF) reliably improved. This has been the first SCED for SO-OCD conducted to date and the first study to evaluate outcomes during CAT using non-linear and non-instantaneous therapeutic change evaluation techniques [11].

CAT was highly effective for all the idiographic measures, bar the control measure, but had the least effect on daily levels of anxiety. Narrative reformulation had an immediate effect on the intensity of worrying and shame (i.e., in comparison to flat and stable baselines). This impact was presumably due to reformulating the SO-OCD as based in the developmental history of the patient. The fastest change, as shown by the steeper lines and larger growth rates in the GLM analyses, were found for reduced worrying, anxiety and shame. CAT had a more gradual effect on the compulsion count, checking and generating evidence measures, as shown by the larger inflection points (i.e., indicating that maximum change occurred later in treatment) and had smaller associated growth rates. Level change was maintained for all the idiographic measures once a plateau was reached. Thus, there was no sign of relapse on any of the idiographic measures.

The Q-LES-Q-SF results showed that CAT enabled the patient to take more enjoyment and satisfaction from his everyday life. In terms of qualitative indices of the effectiveness of the CAT intervention, the patient described feeling more secure in himself beyond his sexual orientation and had learnt that sexual orientation was not a ‘either/or’ concept and that dropping compulsions opened up time in his life for exploring and developing his relationships, both with women and with men. He did not start dating, but said that he was ready to start dating, but still had some anxieties about this. He started to allow himself to enjoy the company and companionship of his male friends, and they had fed back that he had started to be less relationally distant. The patient therefore started to allow himself to enjoy being emotionally intimate with male friends, without doubting this or fearing what this meant. The patient said he still struggled with low mood from time to time and did not want to discontinue his medication. The goodbye letter from the therapist encouraged the many ways in which the patient could embrace life and relationships in the here and now (i.e., without predicting the future) as being a key way of moving away from constantly undermining himself and also a way of developing a more secure sense of identity. In terms of relapse prevention, the goodbye letter emphasised the ‘observing eye’ aspect of CAT—being more aware of the possibility of getting drawn into old patterns and roles, without becoming obsessive about this. The goodbye letter from the patient emphasised the importance of the atmosphere of trust in the therapeutic relationship that enabled him to talk openly and frankly with another man about his sexual orientation fears.

A strength of this study was in the inclusion of a control idiographic measure as this is relatively rare in SCED research. There was a general lack of change in the frequency of intrusions as suggested by the measure of fit and low growth rate, against which the change in other idiographic outcome measures could be compared and contextualised. Although it was a small change, most change occurred in the intrusion count before the intervention began, suggesting that the assessment process did act positively somewhat on the frequency of intrusions. There are limitations to the current study. Given the age of onset of the SO-OCD (18-years), the study could be challenged as merely a young man struggling to construct his identity and then becoming overly focussed on questioning the sexual orientation aspect of that [7]. A formal psychiatric assessment would have confirmed the diagnosis. All data was self-report and so the patient may have felt the need to please the therapist, with outcomes potentially therefore distorted by social desirability response bias. The fact that the idiographic measures were drawn from the SOO-CS (but reworded for daily measurement) could be criticised in terms of not being truly idiographic. The changes observed in these idiographic measures may merely have reflected the changes embedded and recorded in the SOO-CS. The follow-up period was too short to truly assess the long-term effectiveness of the intervention. The design was quasi-experimental as opposed to truly experimental. There was no check on the competency of the treatment delivered. The effect sizes reported (especially ESc) suggest that the greatest intervention effect occurred on the intrusion count idiographic outcome, and this was in contrast to the visual interpretation of the time series and GLM graphs and the nonoverlap statistics.

## 4. Conclusions

A recent scoping review of SO-OCD [32] emphasised how frequently misunderstood this condition is by both patients and clinicians and called for more controlled outcome research to be conducted. This paper therefore makes a significant contribution as it has achieved that. It is very important to differentiate the identification and treatment of SO-OCD with that of so-called ‘conversion therapy’. Conversion therapy refers to the misguided and ineffective attempt to try to change, modify or suppress a patient’s sexual orientation or gender identity. Internationally, a raft of regulatory and legal measures now usefully restricts such practices. This current study of a case of SO-OCD has illustrated the presence of both immediate and gradual non-linear improvements in SO-OCD symptoms occurring through the actions of both the narrative reformulation and then the specific ‘exits’ of CAT during the revision stage of the therapy. This eight-session evaluation suggests that change is possible regarding SO-OCD even with a relatively brief intervention. The current study therefore suggests CAT as an appropriate and effective intervention for SO-OCD that can now be tested in future research with more valid and reliable group designs. Future SCED research with SO-OCD would benefit from using more internally valid experimental designs (e.g., A/B/A/B or ABC) and also generating longer follow-up phases. The relational focus of CAT enables a patient to see how their ‘symptoms’ can be understood and conceptualised in terms of self–self, self–other and other–self relating. SO-OCD is not a commonly presenting problem in clinical services and so the evidence base for intervention could be advanced through the utilisation of more complex single-case methods than was achieved here.

## Figures and Tables

**Table 1:** Idiographic measure descriptives by phase of the study.

	Baseline Phase (2 Sessions)			Treatment Phase (6 Sessions)			Follow-Up Phase		
	Mean	Median	SD	Mean	Median	SD	Mean	Median	SD
Intrusions	16.14	15	2.82	14.23	14	1.63	13.05	13	2.27
Compulsions	15.48	15	3.06	5.21	3	4.91	0.68	0	0.96
Checking	7.57	8	0.68	5.29	5	1.63	3.84	4	0.78
Worrying	9.00	9	0.00	1.54	2	0.54	1.48	1	0.55
Generating evidence	6.48	6	1.69	2.80	2	1.66	0.82	0	0.99
Ashamed	6.86	7	0.65	3.30	3	0.81	2.45	2	0.63
Anxious	8.90	9	0.30	7.77	8	0.76	6.48	6	1.69

**Table 2:** Tau and effect size estimates for idiographic measures between phases.

			Baseline (A) vs. Treatment (B)		
Idiographic Measure	Baseline Trend (τ ^trendA^)	Tau (τ ^AvsB^)	PEM %	NAP %	PAND %
Intrusions	0.057	−0.276 *	66.07	69.90	79.22
Compulsions	0.117	−0.588 *	95.54	94.30	92.21
Checking	0.055	−0.528 *	94.64	88.05	80.52
Worrying	0.000	−0.769 *	100	100	100
Generating evidence	0.110	−0.619 *	96.43	91.75	84.42
Ashamed	−0.037	−0.709 *	100	100	100
Anxious	0.045	−0.588 *	91.07	88.18	84.42
			**Baseline (A) vs. Follow-up (FU)**		
**Idiographic measure**	**Baseline trend** **(τ ^trendA^)**	**Tau (τ ^AvsB^)**	**PEM** **%**	**NAP** **%**	**PAND** **%**
Intrusions	0.057	−0.443 *	78.41	81.28	73.85
Compulsions	0.117	−0.748 *	100	100	100
Checking	10.	−0.742 *	100	100	100
Worrying	0.000	−0.806 *	100	100	100
Generating evidence	0.110	−0.768 *	100	100	100
Ashamed	−0.037	−0.764 *	100	100	100
Anxious	0.045	−0.600 *	90.91	89.50	84.62

* significant between phase difference.

**Table 3:** Model parameters between the GLMs for baseline (A) to intervention (B).

Idiographic Measure	Inflection Point	Growth Rate
Intrusions	20.0	−0.02
Compulsions	25.6	−0.06
Checking	45.8	−0.05
Worrying	21.5	−2.00
Generating evidence	26.6	−0.06
Ashamed	20.1	−0.15

**Table 4:** Model fit and effect sizes for the GLMs on baseline to treatment comparisons.

Idiographic Measure	R^2^	ESc	ESr
Intrusions	−0.39	5.51	1.00
Compulsions	0.78	3.59	1.00
Checking	0.77	3.99	1.00
Worrying	0.97	2.22	0.94
Generating evidence	0.70	3.53	0.92
Ashamed	0.77	2.81	0.83
Anxious	0.54	1.70	0.48

R-squared (R^2^), effect size in relation to Cohen’s D (ESc), and effect size as a proportion (ESr).

**Table 5:** Nomothetic outcomes with associated clinical and reliable change analysis.

	Outcomes			Norms Mean (SD)		RCSI Analysis			
Nomothetic Measure	Assessment Score	Termination Score	Follow-Up Score	Non-Clinical	Clinical	Reliable Change Criteria	Clinical Cut-off	Clinical Change (Y/N)	Reliable Change (Y/N)
PHQ-9	19	8	8	3.3 (3.8)	17.1 (6.1)	>6	≥10	Yes	Yes
SO-OCS	51	12	14	1.36 (3.30)	46.44 (7.98)	6.99	≥18	Yes	Yes
OCI	40	19	17	25.25 (20.8)	66.33 (25.25)	25.01	≥40	Yes	No
Q-LES-Q-SF	43	57	63	54.8 (9.6)	43.64 (9.99)	8.76	-	-	Yes

Blank (-) indicates a lack of norm data to calculate the value. PHQ-9 RCI, clinical and non-clinical norms reported in Kroenke, Spitzer & Williams (2001) [24]. SO-OCS clinical cut-off reported in Melli, Moulding, Gelli, Chiorri and Pinto (2016) [8]. OCI clinical cut-off and clinical and community norms reported in Foa, Kozak, Salkovskis, Coles & Amir (1998) [27]. Q-LES-Q-SF non-clinical norms reported in Arlen et al. (2016) [30] and clinical norms with reliability of the scale reported in Stevanovic (2011) [26].

## Data Availability

Due to privacy concerns, the anonymized data is available on reasonable request from the corresponding author.

## Abbreviations

The following abbreviations are used in this manuscript:

CAT	cognitive analytic therapy
SCED	Single-case experimental design
PEM	percentage exceeding the median
PAND	percentage of all nonoverlapping data
NAP	nonoverlap of all pairs
SOO-CS	Sexual Orientation Obsessive–Compulsive Scale
OCI	Obsessive Compulsive Inventory
PHQ-9	Patient Health Questionnaire-9
GAD-7	General Anxiety Disorder-7
Q-LES-Q-SF	Quality-of-Life Enjoyment and Satisfaction Questionnaire—Short Form
GLM	generalised logistical model
SDR	sequential diagrammatic reformulation
TP	target problem
TPP	target problem procedure
